# Genetic Diversity of *Enterocytozoon bieneusi* in Diarrheic Shelter Dogs in Romania: First Molecular and Phylogenetic Evidence

**DOI:** 10.3390/pathogens14070641

**Published:** 2025-06-27

**Authors:** Mirela Imre, Gheorghe Dărăbuș, Sorin Morariu, Krisztián Szabó, Marius-Stelian Ilie, Tiana Florea, Alexandra Pocinoc, Reem Awwad, Kálmán Imre

**Affiliations:** 1 Faculty of Veterinary Medicine, University of Life Sciences “King Mihai I” from Timisoara, 300645 Timisoara, Romania; gheorghe.darabus@fmvt.ro (G.D.); sorin.morariu@fmvt.ro (S.M.); mariusilie@usvt.ro (M.-S.I.); tijana.florea@usvt.ro (T.F.); alexandra.pocinoc@usvt.ro (A.P.); reem.adver@gmail.com (R.A.); 2 Department of Zoology, Institute for Biology, University of Veterinary Medicine Budapest, H-1077 Budapest, Hungary; szabo.krisztian@univet.hu

**Keywords:** dog, diarrhea, microsporidia, molecular epidemiology, public health

## Abstract

*Enterocytozoon bieneusi* is one of the most common microsporidian parasites, primarily infecting the intestinal epithelial cells of a broad range of animal species, including humans. To date, no scientific reports have documented *Enterocytozoon* spp. in animal hosts in Romania. This study aimed to assess the occurrence and genetic characteristics of *E. bieneusi* in shelter dogs, as well as its potential public health relevance. Between December 2022 and May 2025, a total of 112 freshly voided diarrheal fecal samples were collected from dogs housed in a shelter near Timișoara Municipality, Romania. The samples were subjected to molecular analysis using a two-step nested polymerase chain reaction (PCR) targeting the internal transcribed spacer (ITS) region of the rRNA gene. The resulting sequences were deposited in GenBank^®^ and analyzed phylogenetically. PCR analysis revealed *E. bieneusi* DNA in 11 (9.8%) samples, identifying two genotypes, with PtEb IX (n = 10) as the dominant genotype and BEB4 (n = 1), which has zoonotic potential. A significant difference in prevalence was found between juvenile (23.1%) and adult (5.8%) dogs (*p* = 0.026). Phylogenetic analysis of the ITS sequences showed that the isolates clustered into two distinct clades alongside reference sequences from the GenBank^®^ database. This is the first report of *E. bieneusi* infection in animals in Romania, providing essential baseline data and highlighting the need for broader surveillance into its prevalence and genetic diversity in other potential hosts. These results reflect the prevalence and genetic diversity of *E. bieneusi* exclusively among symptomatic (diarrheic) dogs and should not be generalized to the broader shelter dog population.

## 1. Introduction

Microsporidia are a group of spore-forming obligate intracellular pathogens related to fungi that infect a wide range of vertebrate and invertebrate hosts [1]. Of the more than 1600 recognized species across approximately more than 200 genera, 17 species have been reported to cause infections in humans [2,3]. Among these, the four most common species are *Enterocytozoon bieneusi*, *Encephalitozoon cuniculi*, *E. hellem*, and *E. intestinalis* [4,5]. Of these, *E. bieneusi* is the most prevalent, accounting for more than 90% of microsporidiosis cases in humans [1].

The medical significance of *E. bieneusi* was first recognized in 1985, when it was identified in patients infected with human immunodeficiency virus (HIV) [1]. To date, more than 800 genotypes of this species have been described [6]. These genotypes are classified into 11 major phylogenetic groups, exhibiting varying degrees of host specificity and zoonotic potential [7]. Among them, genotypes A, D, EbpC, and TypeIV, belonging to the largest group, Group 1, are the most commonly detected in human populations. The members of Group 2 are known as ruminant adapted, while Groups 3–11 encompass largely host-adapted genotypes [6].

Microsporidiosis, caused by *E. bieneusi* and transmitted via the fecal–oral route through the ingestion of contaminated food or water containing infective spores, typically presents as an asymptomatic infection or self-limiting diarrhea in individuals with competent immune systems [8]. In immunocompromised individuals (e.g., HIV-positive or cancer patients, the elderly, children, organ transplant recipients, etc.), the pathogen can cause life-threatening and persistent diarrhea [9], with no effective treatment or vaccine currently available.

In addition to humans, *E. bieneusi* has been reported in at least 210 terrestrial species, including dogs (*Canis lupus familiaris*) [6]. The presence of the pathogen in dogs was first reported by Mathis et al. [10] in three individuals from different farms in Switzerland. Since then, more than 20 epidemiological surveys conducted across eight countries (Australia, Columbia, Iran, Japan, Poland, Portugal, Republic of China, and Spain) have confirmed the presence of *E. bieneusi* in various types of dogs (e.g., pets, stray, household, or clinic dogs). These studies recorded an overall prevalence rate of 10.32% (611/5916), with a variable isolation frequency from 0.8% in household dogs to up to 36.9% in stray dogs, according to a review by Jian et al. [11]. In light of this, a considerable number of *E. bieneusi* genotypes have been identified, reflecting a high degree of genetic polymorphism in the internal transcribed spacer (ITS) region of the ribosomal RNA (rRNA) gene [12].

Despite significant advances over the past decade in the study of certain protozoan infections in dogs in Romania, such as *Cryptosporidium* spp. and *Giardia duodenalis* [13,14], no scientific reports have yet been published on the presence of *Enterocytozoon* spp. in any animal host under natural conditions. To address this research gap and in light of the importance of monitoring zoonotic pathogens in companion animals, the present study was undertaken to investigate the molecular prevalence and public health significance of *E. bieneusi* genotypes in diarrheic shelter dogs. Additionally, the study aimed to analyze the distribution of microsporidia in relation to individual animal data and to assess their phylogenetic relationships.

## 2. Materials and Methods

### 2.1. Sample Collection

Given the absence of prior investigations on this topic in Romania, a cross-sectional study design was implemented using a purposive (non-probability) sampling approach to select clinically relevant cases. A total of 112 diarrheic dogs were included between December 2022 and May 2025 from a municipal animal shelter located near (45.7583° N, 21.1122° E) Timișoara Municipality, Romania.

Within the shelter, animals were housed in separate enclosures, either individually or in groups of two to five individuals. The shelter population fluctuated between 150 and 250 dogs during the study period. Sampled dogs were individually selected based on the presence of gastrointestinal symptoms, as identified by the shelter’s veterinary practitioner. Only one sample per dog was collected—no repeated sampling occurred. This sampling methodology is in line with accepted practices for targeted pathogen surveillance in veterinary epidemiology, especially under field constraints, and is consistent with established cross-sectional designs described in the literature [15]. The shelter manager provided informed consent for the inclusion of the dogs in the study and voluntarily contributed fecal samples. The sampling protocol was reviewed and approved by the Bioethics Committee of the University of Life Sciences “King Mihai I” from Timișoara (no. 166/21 December 2022). Freshly voided diarrheic fecal specimens (approximately 20–30 g) were collected from individuals exhibiting gastrointestinal symptoms by a veterinary practitioner responsible for managing animal health at the shelter at various times throughout the study period. Samples were collected immediately after natural defecation without disturbing the dogs using disposable latex gloves, and samples were placed into individually numbered sterile stool containers to ensure proper identification and prevent cross-contamination. They were stored under refrigeration conditions (<4 °C) and transported to the Parasitology Laboratory of the Faculty of Veterinary Medicine, Timișoara, for further analysis. During sampling, individual animal and epidemiological data were recorded, including age (ranging from 1.5 months to 17 years), gender (47 females and 65 males), breed (93 mixed-breed and 19 purebred dogs), and collection season (spring, summer, autumn, or winter). Dogs under 12 months of age were classified as juveniles, while those aged 12 months or older were categorized as adults.

### 2.2. Molecular Analyses

All samples enrolled in the present study were molecularly processed within 48 h of collection. Genomic DNA was extracted from approximately 200 mg of stool using the Isolate Genomic DNA Kit (Bioline Reagents Limited, London, UK), following the manufacturer’s instructions. The extracted DNA was eluted in 100 µL of elution buffer and stored at −20 °C until further processing.

The molecular detection of *E. bieneusi* was performed using a two-step nested polymerase chain reaction (PCR) targeting the internal transcribed spacer (ITS) region of the rRNA gene (~392 bp), following the technique described by Buckholt et al. [16] with minor modifications. The specific primers were EB-F1 forward (5′-GGTCATAGGGATGAAGAG-3′) and EB-R1 reverse (5′-TTCGAGTTCTTTCGCGCTC-3′) for the primary PCR and EB-F2 forward (5′-GCTCTGAATATCTATGGCT-3′) and EB-R2 reverse (5′-ATCGCCGACGGATCCAAGTG-3′) for the secondary PCR. Each PCR was conducted in a total volume of 25 µL, containing 1 µL of each primer (at 10 pmol/µL), 12.5 µL MyTaq™ Red Mix (Bioline Reagents Limited, London, UK), 1 µL of extracted genomic DNA for the primary PCR or 1 µL of the primary PCR product for the secondary PCR, and 10.5 µL ultrapure deionized water. PCRs were performed using the My Cycler Thermocycler (BioRad^®^, Berkeley, CA, USA) with the following cycling conditions: for the primary PCR, an initial denaturation at 95 °C for 1 min, followed by 35 cycles of 94 °C for 30 s (denaturation), 57 °C for 30 s (annealing), and 72 °C for 40 s (extension); both reactions concluded with a final extension step at 72 °C for 10 min. A positive control (DNA from cattle-derived zoonotic genotype CHN1, previously confirmed by sequencing) and a negative control (sterile water) were used in each PCR run. The resulting PCR products were visualized on a 2% agarose gel stained with RedSafe™ (iNtRON Biotechnology, Inc., Gyeonggi-do, Republic of Korea). The 100 bp DNA molecular weight marker (BIOLINE^®^ UK Ltd., London, UK) was used to estimate the size of amplicons.

### 2.3. E. Bieneusi Genotyping and Phylogenetic Analysis

The genotyping of *E. bieneusi* isolates was performed through bidirectional sequencing. All positive secondary PCR products were purified using the Isolate II PCR and Gel Kit (Bioline^TM^ Reagents Limited, London, UK) and sent to Macrogen Europe Company (Amsterdam, The Netherlands) for sequencing. The quality and homology of the resulting sequences were assessed using ChromasPro ver. 2.1.8 (Technelysium Pty Ltd., Tewantin, Australia) DNA sequence analyses software. Subsequently, genotype determinations were performed by comparing the obtained sequences with those available in the GenBank^TM^ database, using the free online Basic Local Alignment Search Tool (BLAST) (available online: https://blast.ncbi.nlm.nih.gov/Blast.cgi, accessed on 15 May 2025). Finally, two representative sequences obtained were deposited in GenBank^®^ as follows: *E. bieneusi* PtEb IX genotype (accession no. PP983236) and *E. bieneusi* BEB4 genotype (accession no. PQ815063).

An analysis of the phylogenetic relationships between the obtained sequences and other GenBank^®^-deposited *E. bieneusi* reference sequences, particularly those isolated from dogs, was conducted based on the internal transcribed spacer (ITS) region of the nuclear ribosomal DNA. Sequence alignment was performed using MAFFT v.7.450 online software (available online: https://mafft.cbrc.jp/alignment/server/, accessed on 15 May 2025) with default settings. After alignment, gaps in the sequences were accounted for and coded as binary characters (present/absent) using the Fastgap 1.2 program, as described by Simmons and Ochoterena [17]. This gap dataset was added as a separate partition to the nucleotide dataset. Models of sequence evolution were assessed using Mega11 [18]. The TN93 + G model was selected for the RNA/ITS region, and uniform rates were applied to the gap dataset. The final dataset included 438 positions (413 nucleotides and 25 gap positions). A maximum likelihood phylogram was constructed using W-IQ-TREE [19], with 1000 ultrafast bootstrap replicates. The resulting phylogram was edited and visualized using the iTOL online platform [20] (available online: https://itol.embl.de/. accessed on 3 March 2025).

### 2.4. Statistical Analysis

*E. bieneusi* infection prevalence was assessed by calculating the proportion of positive results reported to the total number of analyzed samples, along with the corresponding 95% confidence interval. Statistical data interpretation was performed using GraphPad Prism Ver. 10.5.0 (GraphPad Software, San Diego, CA, USA, www.graphpad.com, accessed on 15 May 2025). The chi-squared (χ^2^) test with Yates’ correction was fitted to compare differences in the pathogen distributions in relation to the individual animal data. Differences were considered significant at *p* ≤ 0.05.

## 3. Results

In the present study, the occurrence of *E. bieneusi* in diarrheic dogs was 9.8% (11/112; 95% CI: 5.57–16.73). A sequence analysis of the ITS region of the rRNA gene from the isolates revealed the presence of two genotypes, namely PtEb IX (n = 10) and BEB4 (n = 1). The distribution of *E. bieneusi* genotypes according to the recorded individual animal and epidemiological data is summarized in Table 1. Statistical analysis showed significant differences (*p* = 0.026) in the distribution rates of *E. bieneusi* between juvenile (23.1%; 6/26) and adult (5.8%; 5/86) dogs. No statistically significant association was observed between infection prevalence and season. Similarly, there were no significant differences in infection rates between female (4 out of 43; 8.5%) and male (7 out of 58; 10.8%) dogs or between purebred (2 out of 19; 10.5%) and crossbreed (9 out of 93; 9.7%) dogs, with *p*-values of 0.940 and 0.850, respectively.

All ITS sequences of the *E. bieneusi* PtEb IX genotype recorded in the present study were identical and showed a 99–100% similarity to GenBank^®^ reference sequences MN902239.1 and MK968823.1 (isolated from cats in China), as well as MN046170.1 (isolated from dogs in the Czech Republic). Additionally, the sequence of the *E. bieneusi* BEB4 genotype, isolated from a nine-month-old dog, demonstrated a 99–100% similarity to MT231512.1 and MH732750.1, obtained from calves in China and Ethiopia, respectively.

**Figure 1:**
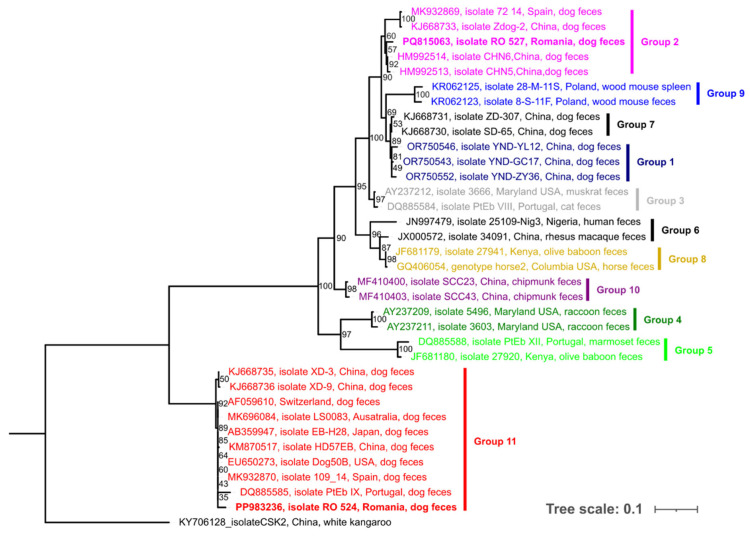
Maximum likelihood phylogram of the internal transcribed spacer (ITS) of the nuclear ribosomal DNA dataset, inferred using W-IQ-TREE, showing the genetic relationships of the *E. bieneusi* genotypes obtained in the present study (bolded) alongside other GenBank^®^-deposited sequences from various hosts and countries. Different colors represent the currently recognized groups (Group 1 to 11) as described in the scientific literature.

Phylogenetic analysis, based on ITS sequences of the rRNA gene, showed that the *E. bieneusi* genotypes, identified in the present study and included in the tree construction, clustered closely with those selected from the GenBank^®^ database and were previously recorded in dogs and other hosts from various countries. The dominant PtEb IX genotype (accession no. PP983236.1) clustered closely with a Portuguese isolate within Group 11, alongside other sequences obtained from dogs in Spain, the United States, China, Japan, Australia, and Switzerland. Additionally, the zoonotic BEB4 genotype (accession no. PQ815063) branched separately in another clade comprising members of Group 2, along with dog origin sequences from China and Spain (Figure 1).

## 4. Discussions

Dogs, as one of humans’ closest companion animals, are well-recognized reservoirs for various zoonotic pathogens due to their close contact with people [1,6]. For this reason, the ongoing data-based health monitoring of dogs, particularly in initiating new investigations into the occurrence of zoonotic pathogens with unknown status in a given region, is both a veterinary and public health priority. To the authors’ knowledge, this is the first published report on the occurrence and molecular characterization of *E. bieneusi* genotypes in a mammalian host in Romania. The findings offer valuable insights for both the country and mainland Europe regarding the distribution, genetic diversity, and potential zoonotic risk of this significant microsporidian species.

In the current investigation, 9.8% (11/112) of the screened diarrheic shelter dogs were found to be infected with *E. bieneusi*. This value is slightly lower than the computed global average prevalence (10.3%) for *E. bieneusi*, reported in various categories of dogs. In this regard, in a representative study, Jian et al. [11] reviewed isolation frequency values reported in available epidemiological investigations, resulting in, in ascending order, the following overall detection rates: 6.5% (36/556) in household dogs, 7.7% (29/376) in dogs from clinics, 7.92% (272/3433) in pet dogs, 8.3% (3/36) in farm dogs, and 17.6% (273/1550) in stray dogs, respectively. However, caution should be exercised when comparing and interpreting these results, as the observed differences may be markedly influenced by characteristics of the study population, such as individual animal factors (e.g., presence or absence of gastrointestinal clinical signs, age categories) and epidemiological variables (e.g., rural or urban origin, applied deworming protocols), as well as geo-climatic conditions, which can affect the environmental survival of microsporidia. It is evident from the available epidemiological data that stray dogs exhibit higher *E. bieneusi* infection rates compared to other dog categories. In the present study, the screened shelter houses provide basic care for homeless or abandoned dogs from the streets of Timișoara and surrounding areas whose epidemiological status was previously unknown. Considering that stray dogs are commonly housed in shelters, and as a comparison point with our study’s infection rate (8.1%), higher prevalence rates have been recorded in China (39.6%–[21]; 20.%–[22]; 18.8%–[23]) and Colombia (15.0%–[24]), while lower rates were reported in Iran (5.3%–[25]), China (8.8%–[26]), and Japan (1.7%–[27]). Given that *E. bieneusi* is regarded as a causative agent of opportunistic infections [1,9], the observed variation in prevalence across studies may be attributed to the current health status of the monitored host populations.

Association analysis revealed that juvenile dogs were significantly associated (*p* < 0.01) with a higher occurrence rate of *E. bieneusi* compared with adult dogs (23.1 vs. 5.8%). Similar findings were reported by Phrompraphai et al. [28] in Japan (8.3% in dogs < 1 year vs. 3.4% in dogs > 1 year) and by Zhang et al. [29] in Australia (2.7% vs. 7.9%), who observed that juvenile animals were 3.11 times more susceptible to *E. bieneusi* infections than adults [29]. Several studies conducted on other livestock [30,31] and humans [29] further support the observation that young age can be considered a risk factor for *E. bineusi* infection.

The analysis of ITS sequence data from the rRNA gene, derived from five samples, revealed the presence of PtEb IX (n = 10) and BEB4 (n = 1) genotypes. The overwhelmingly dominant occurrence of the PtEb IX genotype is not surprising, as it is recognized as a canine-adapted genotype with a relatively narrow host range. To date, this divergent genotype has been reported in dogs, cats, and the European badger [22,32], and it is unlikely to possess infective potential for humans. In contrast, the BEB4 genotype, reported for the first time in dogs in the present study, is considered a ruminant-adapted pathogen, most commonly infecting pre-weaned dairy calves and possessing zoonotic potential. It has previously been reported in two individuals with HIV in the Czech Republic [33]. The detection of the BEB4 genotype in a juvenile dog is noteworthy given its established zoonotic potential. This genotype has been previously isolated from humans, including HIV-positive individuals, indicating potential for opportunistic infection [33]. In the context of shelter environments—characterized by high animal turnover, shared enclosures, and regular human–animal contact—the presence of BEB4 raises concerns for indirect transmission to caregivers or adopters. Enhanced hygiene protocols and molecular screening may help mitigate this risk.

Phylogenetic analysis based on ITS sequence data of the rRNA gene showed that the selected GenBank^®^-deposited *E. bieneusi* isolates clustered into 11 distinct phylogenetic clades. The resulting tree configuration indicated that isolate PP983236 (corresponding to the PtEb IX genotype) clustered within Group 11, along with other GenBank^®^-deposited sequences obtained from dogs. A similar clustering pattern has been previously reported by Zhang et al. [29] and Jian et al. [11], further supporting the increased host specificity of genotypes in this group, which are associated with limited zoonotic potential (reviewed by Li et al. [7]). Another sequence obtained in the present study, namely PQ815063 and representing the BEB4 genotype, clustered within the cattle-hosted Group 2 in the ITS phylogenetic tree. Members of this group are considered zoonotic and are associated with increased cross-species transmission potential [7,11]. Overall, the evolutionary distance analysis confirmed that the ITS sequence region is a reliable and representative genetic marker for defining genetic differences between *E. bieneusi* genotypes. The phylogenetic placement of PtEb IX within Group 11 is consistent with previous studies in dogs across Europe, particularly in Portugal, Spain, and Switzerland [9,10,32]. This supports the hypothesis that PtEb IX is a canine-adapted genotype with limited zoonotic potential. In contrast, the identification of the BEB4 genotype, which clustered in Group 2, aligns with findings in cattle and immunocompromised human patients in Central and Eastern Europe, including the Czech Republic [33]. This highlights the potential for cross-species transmission and underscores the need for the surveillance of genotypes with known zoonotic risk, particularly in urban shelters where animals and humans may interact more closely.

A key limitation of this study is that only dogs exhibiting gastrointestinal symptoms were sampled. As such, the reported 9.8% prevalence reflects the occurrence of *E. bieneusi* among symptomatic dogs and does not represent the true prevalence in the general shelter dog population. Future studies involving randomly selected or asymptomatic animals would be needed to determine overall prevalence. Subsequently, another limitation of this study is the lack of detailed clinical data, such as the severity of diarrheal symptoms and potential co-infections, which could provide further context for interpreting the molecular findings. Future investigations integrating clinical assessments and broader pathogen screening are warranted to better understand the pathogenic role and clinical impact of *E. bieneusi* in dogs.

## 5. Conclusions

This study presents the first molecular evidence of *E. bieneusi* infection in a mammalian host in Romania supported by phylogenetic analysis. The findings suggest that shelter dogs exhibiting gastrointestinal symptoms may act as natural hosts for this pathogen, predominantly carrying the PtEb IX genotype along with the zoonotic BEB4 genotype, which is known to infect both animals and humans. These results, obtained from a region previously lacking epidemiological data, provide valuable insights for veterinary practitioners in diagnosing and managing enteric infections. Furthermore, the study highlights the potential role of shelter dogs in the transmission of microsporidiosis to humans. To better understand the epidemiology of *E. bieneusi* in Romania, further research involving broader sampling and additional molecular investigations in both humans and domestic animals are recommended.

## Figures and Tables

**Table 1:** Distribution of *Entorocytozoon* genotypes in diarrheic dogs according to the recorded data.

Individual Animal and Epidemiological Data	Occurrence [%(n/Total)]	95% Confidence Interval	*E. bieneusi* GenoTypes (No.)	*p*-Value
Age				0.026 *
Juvenile	23.1 (6/26)	11.0–42.1	PtEb IX (6),	
Adult	5.8 (5/86)	25.1–12.9	BEB4, PtEb IX (4)	
Gender				0.940
Female	8.5 (4/47)	3.4–19.9	PtEb IX (4),	
Male	10.8 (7/65)	5.3–20.6	PtEb IX (6), BEB4	
Breed				0.850
Pure	10.5 (2/19)	2.9–3.1	PtEb IX, BEB4	
Crossbreed	9.7 (9/93)	5.2–17.4	PtEb IX (9)	
Season				0.707
Spring	12.1 (4/33)	4.8–27.3	PtEb IX (3), BEB4	
Summer	5.9 (1/17)	1.1–27.0	PtEb IX	
Autumn	5.0 (1/20)	0.9–23.6	PtEb IX	
Winter	11.9 (5/42)	5.2–25.0	PtEb IX (5)	

* = statistically significant difference (*p* < 0.05) computed by using the chi-squared (χ^2^) test with Yates’ correction.

## Data Availability

The data are contained within the article.
